# The Association Between Malaria Parasite Geometrical Mean and Clinical Spectrum of Severe Disease in a High-Transmission Setting in Eastern Uganda: A Cross-Sectional Study

**DOI:** 10.1155/japr/4801721

**Published:** 2025-06-02

**Authors:** Emma Isaiah Eregu Egiru, Cate Namayanja, George Paasi, William Okiror, Paul Ongodia, Charles Benard Okalebo, Rita Muhindo, Grace Abongo, Faith Oguttu, Ambrose Okibure, Francis Okello, Crispus Tegu, David Mukunya, Martin Chebet, Peter Olupot-Olupot

**Affiliations:** ^1^Clinical Trials Unit, Mbale Clinical Research Institute, Mbale, Uganda; ^2^Department of Pediatrics and Child Health, Busitema University Faculty of Health Sciences, Mbale, Uganda; ^3^Department of Community and Public Health, Busitema University Faculty of Health Sciences, Mbale, Uganda; ^4^Department of Research, Nikao Medical Center, Kampala, Uganda; ^5^Varimetrics Group Limited, Mbale, Uganda

## Abstract

**Background:** Malaria burden remains significant, especially in high-transmission settings. While some data show an association between severe malaria and high-malaria parasite geometrical mean (GM), few data describe this phenomenon in malaria high-transmission settings. We described the malaria parasite GM and clinical spectrum of severe malaria in Eastern Uganda to advance understanding of its implications on disease severity and patient outcomes.

**Methods:** We conducted a cross-sectional study in Mbale Regional Referral Hospital (MRRH), Eastern Uganda. Children admitted with severe malaria confirmed by microscopy with ages between 2 months and 12 years were enrolled in the study from September 21, 2021, to September 21, 2022. Data were collected on patient sociodemographics, clinical symptoms and signs, laboratory parameters, treatment details, and outcomes. From the blood samples collected at the bedside, blood films/smears were made. The malaria parasite count was obtained from the patients' smears by counting the malaria parasites against 200 white blood cells (WBCs). The GMs of malaria were obtained after the logarithmic transformation of the parasite counts. Data were analyzed using Stata 15, and significant associations were reported at *p* values of 0.05 at 95% confidence intervals.

**Results:** A total of 376 children with a mean age of 4.65 years were recruited, of whom 57.71% (217/376) were male. Children under 5 years constituted 61.7% (232/376). The common clinical manifestations were prostration 76.9% (289/376), jaundice 55.6% (209/376), severe anemia 48.4% (182/377), and hemoglobinuria 46.5% (175/376). The overall malaria parasite GM was 12,238.42 parasites/microliter (95% CI: 9166.72–16,339.43). The highest GM of 197,000 parasites/microliter (95% CI: 40,817.64–946,368) and the lowest of 8938.185 parasites/microliter (95% CI: 5932.8–13,466.01) were observed in shock and severe anemia, respectively. Inpatient mortality was 3.4%.

**Conclusion:** In malaria high-transmission settings of Eastern Uganda, patients with severe malaria had low parasite GMs similar to those in uncomplicated malaria. Thus, malaria parasite GM should not be relied upon to determine disease severity in these settings.

## 1. Introduction

Infection with malaria is still rampant in sub-Saharan Africa (SSA). In 2022, SSA accounted for 93.6% of 249 million malaria cases globally [1]. Only four countries in SSA contributed significantly to the global malaria burden in 2022 notably Nigeria (26.8%), the Democratic Republic of the Congo (12.3%), Uganda (5.1%), and Mozambique (4.2%) [1]. While data before 2010 suggested that high parasitemia played a role in influencing malaria severity, morbidity, and mortality [1–7], few data, however, have reported on malaria parasite geometrical mean (GM) with disease severity. Against this background, the overall picture of the malaria parasite GM and its relationship with disease severity remains incomplete. Earlier studies suggest a lower parasite GM is associated with severe clinical manifestations [1, 4–8], while others indicate that high GM does not necessarily correlate with disease severity [9–12]. This dichotomy may be attributed to differences in disease endemicity and transmission patterns. For instance, studies reporting higher GM without associated complications were conducted in areas with, or during times of high disease transmission [9–14]. Conversely, data reporting lower GM with associated complications were observed in settings with or during periods of low malaria transmission [1, 4–8]. Limited data have described malaria parasite GM levels with specific clinical forms in high-transmission settings, though such information has the potential to inform clinical practice and research. Malaria parasite GM is not directly included as a criterion for severity in the WHO severe malaria surveillance criteria which encompasses both clinical and laboratory parameters [1, 8]. This could be because of the cumbersome process requiring statistical analyses that would not be readily available in most settings. Published data report malaria parasitemia in various forms including malaria parasite density (PD), which is a quantitative measure of the number of parasites in a given volume of blood [7, 8, 15]. In addition, hyperparasitemia, which refers to an abnormally high PD (> 250,000/*μ*L) is included as one of the WHO criteria for classifying severe malaria [1, 6–8]. The determination of hyperparasitemia is context-dependent and may involve considering both the percentage of infected red blood cells (> 10%) and/or the absolute number of parasites [1, 6–8]. Lastly, malaria parasite GM is a measure of central tendency that is calculated by multiplying together a set of values and then taking the *n*^th^ root of the product, where *n* is the number of values [16, 17]. The GM is useful when dealing with data that spans multiple orders of magnitude, such as parasite counts in malaria [16–18]. It provides a more accurate representation of the typical value of the parasite counts. Despite this perceived usefulness, malaria parasite GM is not frequently calculated or reported, especially outside of the research settings. It involves obtaining a set of malaria parasite counts from individual patients, getting the arithmetic means, and using logarithmic transformation of these arithmetic means to acquire the GMs [16–18]. There are hardly any reports on the use of GM for informing clinical care. Some reports, however, have published data on GM for predicted patient outcomes [2, 4, 10, 19, 20].

Studies in Eastern Uganda have reported associations between low parasitemia and severe anemia and Blackwater fever (BWF), while high parasitemia is associated with cerebral malaria [12, 14, 21]. However, formal descriptions of associations between parasite GM and the clinical spectra of severe malaria in this context are lacking. This study is aimed at determining the association between the malaria parasites GM in patients with severe malaria in Eastern Uganda.

## 2. Methods

### 2.1. Study Design and Setting

**Figure 1:**
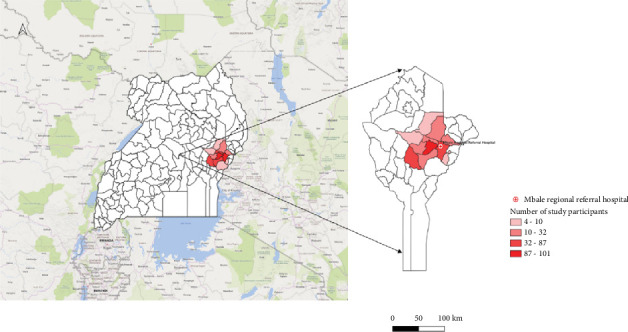
Map of Eastern Uganda showing the catchment areas for this study.

This was a cross-sectional study under the Strengthening Malaria Epidemiological, Pathophysiological, and Intervention studies in Highly Endemic Eastern Uganda (TMA 2016SF-1514-MEPIE Study), and was conducted at the Pediatric Acute Care Unit (PACU) of Mbale Regional Referral Hospital (MRRH) in Mbale, Eastern Uganda, spanning from September 21, 2021, to September 21, 2022. The PACU attends to all children reporting to MRRH with acute illnesses within the age range of 60 days (2 months) to 12 years.  Figure 1 shows the catchment areas in Eastern Uganda where the study was carried out.

### 2.2. Study Procedures

#### 2.2.1. Study Participants

Children aged between 2 months and 12 years with malaria, confirmed by microscopy, and exhibiting one or more clinical and/or laboratory features of severe malaria as defined by WHO [1, 8] with consent from their caretakers/parents were eligible for the study.

#### 2.2.2. Study Phenotypic Data

Data were collected using a customized proforma on social demographics, clinical presentation, treatment, and outcomes.

#### 2.2.3. Inclusion and Exclusion Criteria

Using the WHO criteria definitions, we included children with prostration (inability to sit, stand, or walk without assistance due to generalized weakness), impaired consciousness (Blantyre coma score < 3), multiple convulsions (2 or more convulsions in 24 h), severe anemia (Hb less than 5 g/dL), pulmonary edema/respiratory distress (Kussmaul's breathing manifesting as deep breathing with signs of increased work of breathing, shock (compensated shock with capillary refill time ≥ 3 s or temperature gradient and no hypotension, decompensated shock with systolic blood pressure < 70 mmHg in children with evidence of impaired perfusion like cool peripheries or prolonged capillary refill), spontaneous hemorrhage (recurrent or prolonged bleeding from nose gums or venipuncture sites; hematemesis or melena), hemoglobinuria, jaundice (plasma bilirubin > 50 umol/L (> 3 mg/dL)), hypoglycemia (< 2.2 mmol/L or < 40 mg/dL), acidosis (a plasma bicarbonate concentration < 15 mmol/L or base excess below −8 meq/L), lactic acidosis (plasma lactate > 5 mmol/L), hyperparasitemia (250,000 parasites/microliter or greater than 10% of RBCs), renal failure (serum creatinine 265 *μ*mol/L (> 3 mg/dL)/blood urea > 20 mmol/L (122 mg/dL), or BUN 20 mmol/L (57 mg/dL)) [8, 12, 14].

For sample size estimation, a formula for cross-sectional studies by Kish and Leslie (*n* = (*Z*^2^ pq)/*e*^2^) was used to estimate the sample size using *p* as the prevalence of hyperparasitemia in severe childhood malaria [22]. The prevalence of hyperparasitemia, *p*, was taken as 62.4% from Olupot-Olupot et al. [12].

Children above or below the age bracket (2 months to 12 years), without malaria confirmation, and whose parents did not consent and/or did not fulfill the WHO severe malaria criteria 2014 were excluded from the study.

### 2.3. Laboratory Procedures

The laboratory analyses encompassed a comprehensive array of tests, including complete blood counts, lactate, glucose, blood gases, electrolytes, metabolites, and coagulation markers. Blood smears were obtained and subjected to a methanol-stabilized Romanosky stain, at pH 7.2 (Giemsa 10% stain), to identify, quantify, and categorize *Plasmodium* parasites. Microscopic examination under x400 and x1000 objectives was conducted. Malaria parasites were quantified by counting against 200 white blood cells (WBCs) under the x100 objective [7, 8, 15].

### 2.4. Computation of Malaria Parasite GMs

We obtained the malaria parasite count from the patients' samples (from their blood films/smears).

Natural logarithms (base *e*) of each parasite count were then calculated using the logarithm of count = ln (parasite count). This was to linearize the data and make it suitable for calculating the GM [16, 17, 23, 24].

The arithmetic mean of logarithms was calculated by summing up all the individual logarithms and dividing them by the total number of counts as represented in the formula: arithmetic mean of logarithms = ∑(ln (parasite count))/number of counts [16, 17, 23, 24].

Then, the GM was calculated last using the arithmetic means (mean log transformation) by raising *e* (the base of the natural logarithm) to the power of the arithmetic mean obtained in the previous step. Denoted by the following: GM = *e* (arithmetic mean of logarithms) [16, 17, 23, 24].

For quality control in obtaining the parasite count, the smears were read by two microscopists. A third microscopist was requested to read the smear in case of any discrepancies, and their result was taken as the tiebreaker.

### 2.5. Data Analyses

A formula for cross-sectional studies by Kish and Leslie was used to estimate the sample size using p as the prevalence of hyperparasitemia in severe childhood malaria [22]. The prevalence of hyperparasitemia was taken as 62.4% from Olupot-Olupot et al. [12].

Data were summarized using frequency tables, charts, and proportions. Continuous variables were displayed using means, median, standard deviation (SD), and interquartile ranges (IQRs). Both nominal and categorical variables were represented using numbers, charts, and percentages.

A Kruskal–Wallis test was conducted to compare the GMs among children presenting with one, 2–4, and more than four clinical phenotypes. Additionally, a Mann–Whitney test was performed to investigate associations between the GMs and the different malaria forms. Significance was determined using 95% confidence intervals, with *p* values of less than 0.05 considered significant.

Before data collection, ethical approval was obtained from the Mbale Regional Referral Hospital Research Ethics Committee (MRRH-REC) under the number MRRH-2021-77.

## 3. Results

### 3.1. Demographics

A total of 897 children were screened, and 376 eligible patients were recruited, with a male-to-female ratio of 1.36. The mean age of the participants was 4.65 years, with the youngest child diagnosed with severe malaria being 2 months old. Children under 5 years old accounted for 61.7% (232/376) of the recruited sample (Table 1).

### 3.2. Severe Malaria Forms

The most prevalent forms of severe malaria were as follows: prostration 76.9% (289/376), jaundice 55.6% (209/376), severe malarial anemia 48.4% (182/377), and hemoglobinuria 46.5% (175/376). Cerebral malaria accounted for 7.5% (28/376), impaired consciousness 23.1% (87/376), respiratory distress 8.2% (31/376), acidosis 15.4% (58/376), and renal failure13.6% (51/376).

**Figure 2:**
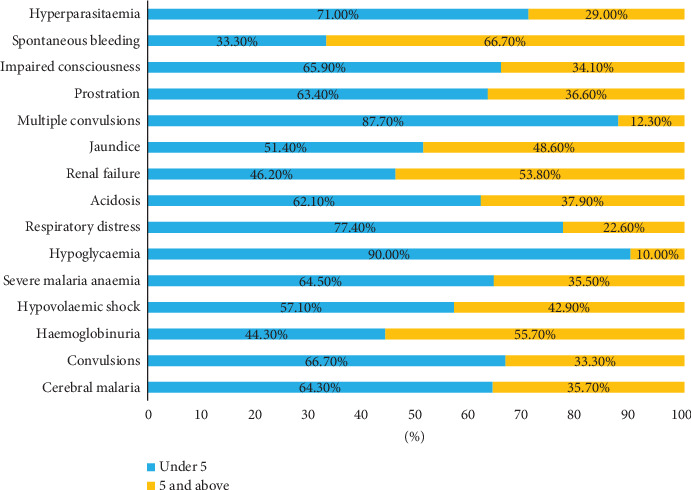
Graph showing the distribution of clinical forms of severe malaria along the age groups.

Figure 2 shows the distribution of severe malaria forms with age.

### 3.3. GM

The GM for children with more than 4 severe malaria forms (14,562.8 parasites/microliter (95% CI: 8170.4–25,956.5)) was higher than those with 2–4 severe malaria forms (13,388.3 parasites/microliter (95% CI: 9316.3–19,240)) and 1 severe malaria form (5183.3 parasites/microliter (95% CI: 2158.1–12,448.9)) (Table 2). Children under 5 years of age had a higher GM compared to those over 5 years, with values of 16,704.33 parasites/microliter (95% CI: 11,418.32–24,437.47) versus 7414.094 parasites/microliter (95% CI: 4807.443–11,434.1), resulting in a geometric mean ratio of 0.44 (95% CI: 0.25–0.79); *p* = 0.006 (Table 3).

The highest and lowest GM were observed in patients with shock 197,000 parasites/microliter (95% CI: 40,817.64–946,368) and severe malarial anemia 8938.185 parasites/microliter (95% CI: 5932.8–13,466.01), respectively. The GMs for other manifestations were as follows: cerebral malaria 12,493.58 parasites/microliter (95% CI: 4287.426–36,406.33), multiple convulsions 19,520.39 parasites/microliter (95% CI: 9493.525–40,137.41), prostration 13,366.4 parasites/microliter (95% CI: 9609.92–18,591.28), respiratory distress 16,800.58 parasites/microliter (95% CI: 5987.739–47,139.58), and acidosis 12,729.4 parasites/microliter (95% CI: 6,126.089–26,450.43).

The predominant species identified was *Plasmodium falciparum (Pf)*, with only one patient presenting with mixed malaria species (*Pf and P. ovale*). In terms of chronic illnesses, 4.3% (16/367) of the patients had sickle cell anemia, and 1.9% (7/367) were found to be infected with HIV.

### 3.4. GM for Children With Different Numbers of Clinical Forms of Malaria

On admission, 63% (237/376) of the children presented with 2–4 severe malaria forms, while 11.4% (43/376) presented with only one severe malaria form. The GM for children with 2–4 syndromes was 13,388.3 parasites/microliter (95% CI: 9316.3–19,240), while it was 14,562.8 parasites/microliter (95% CI: 8,170.4–25,956.5) for children with more than four syndromes (Table 1). The results from the Kruskal–Wallis test (*χ*^2^ = 274.4, **p** < 0.0001) indicated a statistically significant difference in the GM among children presenting with one, 2–4, and more than four clinical forms of severe malaria. Among the patients who unfortunately succumbed to the disease, 75% (9/12) had more than four clinical syndromes (Table 2).

### 3.5. Geometric Mean Ratios (GMRs) for Children With Different Clinical Forms of Malaria

Patients with 2–4 malaria clinical syndromes had a GMR of 2.58 (95% CI: 1.03–6.50) compared to those with one syndrome, while patients with more than four malaria clinical syndromes had a GMR of 2.81 (95% CI: 1.01–7.81) compared to those with one syndrome. This is detailed in Table 3.

Regarding specific clinical conditions, patients with hypovolemic shock had a GMR of 16.93 (95% CI: 5.06–56.66) compared to those without hypovolemic shock. Patients with cerebral malaria showed a GMR of 1.02 (95% CI: 0.36–2.93) compared to patients without cerebral malaria. For patients with severe anemia, their GMR was 0.54 (95% CI: 0.32–0.97) compared to those without severe anemia. Patients with hemoglobinuria had a GMR of 0.58 (95% CI: 0.32–1.02) compared to those without hemoglobinuria. Those with multiple convulsions had a GMR of 1.78 (95% CI: 0.82–3.87) compared to patients without multiple convulsions. Patients aged above 5 years had a GMR of 0.44 (95% CI: 0.25–0.79) compared to those under 5 years (Table 3).

The GM ratio for patients who died was 0.94 (0.25–3.51) compared to patients who survived.

The GM for patients who died was 11,213.4 parasites/microliter (95% CI: 2501.6–50,264.8). Among the deceased, 75% (9/12) had more than four clinical syndromes, and the GMR for those who died was 0.94 (95% CI: 0.25–3.51); *p* = 0.921.

The in-hospital mortality rate was 12 out of 357, accounting for 3.4% (CI: 1.3–5.5).

Mortality was associated with acidosis *p* = 0.0748 (Table 4).

## 4. Discussion

Eastern Uganda is characterized by high malaria transmission. The region recently experienced a malaria epidemic from June 2022 to May 2023 [25, 26]. Data describing GM in the various malaria clinical spectra are limited; however, such data would provide valuable insights into whether they predict patient severity and outcomes or are useful for informing the design of future research. For example, standard malaria rapid diagnostic tests (mRDT) frequently miss low-density *Pf* [1, 8, 20, 27], leading to potential delays in initiating appropriate treatment and resulting in poor outcomes. This study has demonstrated that in the malaria-endemic area of Eastern Uganda, the malaria parasite GM, both the overall and for the specific different severe malaria clinical spectra in children, were lower compared to similar data reported elsewhere [12, 19, 28, 29]. Could this observation represent a novel phenomenon reporting low parasite GMs in unusual clinical spectra of severe malaria in high-transmission settings in the epidemic-prone region of Eastern Uganda? [14]. Possibly, but more research in this area is needed cognizant of the unpredictable nature of malaria epidemiology and transmission patterns.

Considering the pathophysiology of severe malaria, factors such as sequestration of malaria parasite outside the vascular system and in organs, inept and low immune response secondary to available malaria prevention strategies and interventions, and/or a decline in immunity before the malaria epidemic [1, 5, 13, 19, 27, 30] could explain the observed low GMs in severe malaria in some settings. Furthermore, the initiation of antimalarial medications before the estimation of parasitemia may have possibly played a role.

Earlier data on severe malaria in the same setting reported a GM of 58,800 parasites/microliter [12], while data from the central part of Uganda reported 95,433 parasites/microliter [19]. Our study, in severe malaria, posted a GM of 12,238.42 parasites/microliter which is lower than 58,800 parasites/microliter [12] and 95,433 parasites/microliter [19] reported by Olupot et al. and Idro et al, respectively, on severe malaria. The differences could be attributed to several factors, including but not limited to the timing of the studies. This study was conducted during the post-COVID-19 period, which coincided with the malaria epidemic [25, 26]. During this period, it was observed that few, specifically the very critically sick, accessed health services (referral services) in all facilities nationwide, limited numbers of health workers were at their workstations, and there were disruptions in the delivery and acquisition of drug supplies due to the lockdown effect of COVID-19 management protocols. The same effect could have contributed to the occurrence of the malaria epidemic. It is a possibility given that before COVID-19, malaria prevention interventions were well implemented, but suddenly, these measures were not adhered to during the COVID-19 period, thus abruptly exposing the community to malaria.

This study has also shown that low GMs are not synonymous with uncomplicated malaria. They can be observed in complicated/severe malaria. As noted above, our study, in severe malaria, posted a GM lower than that reported by Olupot et al. and Idro et al. on severe malaria. There are possibilities, singly or in combination, which could explain this, including parasite sequestration outside the vascular system, hence low peripheral circulation of the parasites, prior treatment with antimalarial medications before seeking care, and the utilization of the intermittent prevention and treatment (IPT) of malaria services, which could alter the malaria syndromes observed and the level of peripheral parasitemia obtained. On the flip side, several studies on uncomplicated malaria have reported higher or relatively similar GMs to the one reported in this study. For instance, Muhindo et al. reported a GM of 13,258 parasites/microliter in 2014 [28], Lehane et al. reported a GM of 18,996 parasites/microliter in 2019 [29] and Kakande et al. reported a GM of 11,400 parasites/microliter in 2020 [31]. Compared to the GM in uncomplicated malaria, the GM in this study was 12,238.42 parasites/microliter which is lower than 18,996 parasites/microliter and 13,258 parasites/microliter reported by Lehane et al. and Muhindo et al, respectively, on uncomplicated malaria. These data further indicate that based on the parasite GM alone, it is difficult to classify severe malaria since similar and higher readings are reported among patients with uncomplicated malaria.

The most observed forms of severe malaria were prostration, jaundice, severe malarial anemia, and hemoglobinuria. Higher GMs were noted in patients with hypovolemic shock and multiple convulsions, suggesting a possible pseudohigh parasitemia secondary to decreased plasma volume and parasite sequestration, respectively. Data from other sources observed that higher GMs were reported in patients with cerebral malaria, and impaired consciousness [8, 19, 32]. For instance, Idro et al. reported an association of a high parasite GM in young children with seizures in areas of high transmission intensity [32]. Similarly, we observed higher GMs in patients with cerebral malaria, multiple convulsions, and impaired consciousness. In addition, severe malaria patients with cerebral manifestations (cerebral malaria, multiple convulsions, and impaired consciousness) had higher GMRs compared to patients without cerebral manifestations. It is possible that sequestration of the malaria parasites in the brain was responsible for the low GMs in patients with cerebral manifestations than the thresholds previously reported [8]. Additionally, all the malarial syndromes' in this study had low GMs.

Host factors, parasite adaptability, age, and transmission intensity have been identified as contributing factors to malaria severity [13, 19, 33, 34]. Younger children, especially those under five, had a higher GM compared to children older than 5 years of age, suggesting the role of underdeveloped immune systems in the former. A large proportion (46%) of the patients reported the use of antimalarial drugs before admission with severe malaria. Whether the antimalarial medications used in the community were not effective, the dosing used was inappropriate, or the emerging phenomenon of malaria drug resistance and/or tolerance was responsible for the severity of disease with low GM, this could not be established in this study.

Children presenting with more than four malaria syndromes were sicker and had higher GMs compared to those with fewer than four malaria syndromes. This aligns with previous studies demonstrating an increase in disease severity, morbidity, and mortality in patients presenting with more severe features [10, 12, 13, 19, 35]. In-hospital mortality was 12/357 (3.4%, 95% CI: 1.3–5.5). Mortality was associated with deep acidotic breathing (*p* = 0.0748), consistent with other series on severe malaria [13, 14, 35]. Children with more than four severity syndromes accounted for 75% (9/12) of all mortality.

### 4.1. The Study Had some Limitations

For instance, there were no baseline data on threshold levels of malaria parasite GMs premalaria epidemic and pre-COVID-19 periods for comparison purposes. In addition, it was challenging to determine the role of preadmission antimalarial treatment, including the efficacy of those medications in the causation of low parasite GMs in these patients. The study did not measure the immune levels of the study participants, and unfortunately, 19 patients self-discharged, and as a result, their outcomes were not captured. Nonetheless, this study has shown that a low GM can be present in severe malaria and that parasitemia alone is not a good criterion for classifying severity.

## 5. Conclusion

Low GM of malaria parasites was observed across various clinical spectra of severe malaria in this study, similar to the GMs in uncomplicated malaria. Children under 5 years were noted to have higher GM compared to those over 5 years. Malaria parasite GM proves ineffective as a classification criterion for severe malaria because it fails to differentiate it from uncomplicated malaria and thus should not be relied upon to determine disease severity.

## Figures and Tables

**Table 1:** Parasite geometric mean of children with different forms of severe malaria.

**Category**	**Frequency**	**Percentage (%)**	**Geometric mean (95% CI) (parasites/*μ*L)**
*Malaria classification*			
Cerebral malaria	28	7.5	12,493.58 (4287.426–36,406.33)
Hemoglobinuria	175	46.5	9114.115 (6079.518–13,663.43)
Hypovolemic shock	7	1.9	197,000 (40,817.64–946,368)
Severe malaria anemia	182	48.4	8938.185 (5932.8–13,466.01)
Respiratory distress	31	8.2	16,800.58 (5987.739–47,139.58)
Acidosis	58	15.4	12,729.4 (6126.089–26,450.43)
Renal failure	51	13.6	8957.945 (4098.774–19,577.75)
Jaundice	209	55.6	11,482.53 (7869.867–16,753.59)
Multiple convulsions	73	19.4	19,520.39 (9493.525–40,137.41)
Prostration	289	76.9	13,366.4 (9609.92–18,591.28)
Impaired consciousness	87	23.1	13,397.96 (7291.555–24,618.27)
Spontaneous bleeding	6	1.6	17,312.04 (2060.311–145,000)
Overall (mean)	376	100	12,238.4 (9166.7–16,339.4)
*Age*			
Under 5 years	232	61.7	16,704.33 (11,418.32–24,437.47)
5 years and above	144	38.3	7414.094 (4807.443–11,434.1)
*Number of malaria clinical syndromes*			
1	44	11.7	5183.3 (2158.1–12,448.9)
2–4	237	63	13,388.28 (9316.349–19,239.95)
> 4	95	25.3	14,562.82 (8170.433–25,956.5)
*Outcomes*			
Alive	344	96.6	11,987.5 (8837–16,261.1)
Dead	12	3.4	11,213.4 (2501–50,264.8)

**Table 2:** Geometric mean for children with different numbers of clinical forms of malaria.

**Number of malaria syndromes**	**Number**	**Geo. mean (parasites/*μ*L)**	**Min (lower 95%) (parasites/*μ*L)**	**Max (upper 95%) (parasites/*μ*L)**
Syndromes				
1	44	5183.3	2158.1	12,448.9
2 ≤ 4	237	13,388.3	9316.3	19,240
> 4	95	14,562.8	8170.4	25,956.5
Overall	376	12,238.4	9166.7	16,339.4
Alive				
1	43	5426.4	2222.9	13,246.1
2 ≤ 4	220	12,432.1	8505.1	18,172.3
> 4	81	16,538.7	8766.8	31,200.5
Overall	344	11,987.5	8837	16,261.1
Dead				
1		0	0	0
2 ≤ 4	3	94,822.5	32,373.7	277,734.5
> 4	9	5504	931.7	32,516.1
Overall	12	11,213.4	2501.6	50,264.8

**Table 3:** Geometric mean ratios (GMR) for children with the different clinical forms of malaria.

**Category**	**Frequency**	**Percentage**	**Geometric mean ratio (GMR)**	**p** ** value**
Number of malaria clinical syndromes				
1	44	11.7	1	—
2–4	237	63.0	2.58 (1.03–6.50)	0.044
> 4	95	25.3	2.81 (1.01–7.81)	0.048
Malaria classification				
Cerebral malaria	28	7.5	1.02 (0.36–2.93)	0.967
Hemoglobinuria	175	46.5	0.58 (0.32–1.02)	0.06
Hypovolemic shock	7	1.9	16.93 (5.06–56.66)	< 0.001
Severe malaria anemia	182	48.4	0.54 (0.31–0.97)	0.038
Respiratory distress	31	8.2	1.41 (0.51–3.94)	0.508
Acidosis	58	15.4	1.05 (0.48–2.29)	0.907
Renal failure	51	13.6	0.7 (0.31–1.59)	0.388
Jaundice	209	55.6	0.87 (0.48–1.56)	0.631
Multiple convulsion	73	19.4	1.78 (0.82–3.87)	0.142
Prostration	289	76.9	1.46 (0.74–2.90)	0.274
Impaired consciousness	87	23.1	1.12 (0.57–2.23)	0.735
Spontaneous bleeding	6	1.6	1.42 (0.31–6.50)	0.648
Age				
Below 5 years	232	61.7	1	—
5 years and above	144	38.3	0.44 (0.25–0.79)	0.006
Outcomes				
Alive	344	96.6	1	—
Dead	12	3.6	0.94 (0.25-3.51)	0.921

**Table 4:** Association of geometrical mean with mortality in severe malaria spectrum.

**Severe malaria classify**	**Geometrical mean (*n*)**			**% mortality**	**p** ** value**
	**All samples (parasites/*μ*L)**	**Died (parasites/*μ*L)**	**Alive (parasites/*μ*L)**		
Cerebral malaria	10,341.2 (28)	21,420 (5)	2384.8 (21)	17.9	0.9740
Hemoglobinuria	17,600 (176)	15,160 (4)	18,400 (163)	2.3	0.7377
Hypovolemic shock	274,380 (7)	0.0 (0)	274,380 (7)	0.0	—
Severe malaria anemia	8900 (183)	3552.5 (8)	11,300 (163)	4.4	0.3761
Hypoglycemia	55,620 (20)	4542.0 (1)	94,980 (19)	5.0	0.4652
Respiratory distress	21,420 (31)	2864.5 (6)	24,640 (23)	19.4	0.1185
Acidosis	21,210 (58)	2864.5 (6)	25,200 (49)	10.3	0.0748
Renal failure	7381.05 (50)	4542.5 (5)	25,200 (45)	9.4	0.6391
Jaundice	21,710 (210)	4542.5 (7)	22,000 (193)	3.3	0.2742
Multiple convulsions	57,600 (73)	125,120 (5)	72,489.99 (64)	6.8	0.8170
Prostration	26,410 (290)	8900 (11)	27,440 (261)	3.8	0.5960
Impaired consciousness	19,770 (88)	21,420 (11)	9050 (72)	12.5	0.7657

## Data Availability

The data that support the findings of this study are available from the corresponding author upon reasonable request.
